# Quality of Informed Consent Practices around the Time of Childbirth: A Cross-Sectional Study in Italy

**DOI:** 10.3390/ijerph19127166

**Published:** 2022-06-10

**Authors:** Emanuelle Pessa Valente, Ilaria Mariani, Benedetta Covi, Marzia Lazzerini

**Affiliations:** Institute for Maternal and Child Health, IRCCS “Burlo Garofolo”, WHO Collaborating Centre for Maternal and Child Health, Via dell’Istria 65/1, 34137 Trieste, Italy; ilaria.mariani@burlo.trieste.it (I.M.); benedetta.covi@burlo.trieste.it (B.C.); marzia.lazzerini@burlo.trieste.it (M.L.)

**Keywords:** informed consent, childbirth, WHO standards, healthcare provider, quality of care, quality improvement

## Abstract

Background: Few studies have explored consent request practices during childbirth. Objective: We explored consent request practices during childbirth in a referral hospital and research centre in Italy, capturing both women and health workers’ perspectives. Methods: Data were collected using self-administrated questionnaires between December 2016 and September 2018. Nine key maternal and newborn procedures were analysed. Associations between consent requests and women characteristics were explored by multiple logistic regression. Results: Among 1244 women, the rate of consent requests varied widely, with caesarean section (CS) showing the highest rate (89.1%) and neonatal conjunctivitis prophylaxis presenting the lowest rate (11.4%). Information provided on “risks/benefits” and “reasons” for procedures by health staff was most often not comprehensive for procedures of interest (range 18.6–87.4%). The lack of informed consent is not specifically linked to any pattern of women characteristics. According to 105 health workers, adequate protocols and standard forms for consent requests were available in 67.6% and 78.1% of cases, respectively, while less than one third (31.4%) reported having received adequate training and supportive supervision on how to deliver informed consent. Conclusions: Study findings align with previous evidence showing that consent request practices during childbirth need to be largely improved. More research is needed to investigate effective strategies for improvement.

## 1. Introduction

‘Informed consent’ is the intentional communication process where benefits, risks and alternatives of a treatment or procedure are disclosed, allowing independent acceptance or rejection by patients on the basis of their own preferences, personal values or goals [1,2,3]. Its main objective is to promote autonomous decision making during research or during clinical practice [4]. Women’s information and consent to care during childbirth are key principles for women-centred care [5,6,7], but also a fundamental right of women and newborns [8,9,10,11,12], and a legal requirement in most countries.

The procedures related to consent request may vary depending on the setting and local regulations. Written informed consent is formally needed for research purposes, although in real settings this is not always performed. A secondary analysis of a Cochrane network meta-analysis reported that out of 192 studies reporting data for 133,793 women on post-partum haemorrhage (PPH) prevention, in only 59.9% of them, women informed consent was sought at admission for childbirth [13]. 

Considering clinical practice, there is little information available on consent request around the time of childbirth in the WHO European region [14,15,16,17,18], in particular women and health worker’s combined perspectives are lacking. A large multi-country survey among 21,027 women in 12 European countries reported gaps in consent request, with a large heterogeneity in practices: 34.7% of women did not feel involved in choices related to the medical interventions received (21.7% in Luxemburg to 77.2% in Serbia) and, among those who underwent labour, 53.6% did not provide consent for an instrumental vaginal birth (35.9% in Sweden to 81.8% in Croatia) [15]. These data align with those from low- and middle-income countries [19,20,21,22,23]. For example, during 253 continuous observations of labour/childbirth in four African countries, 75.1% of women did not consent to episiotomy, despite receiving it [21]. A systematic review including 15 studies reported that in Ethiopia, 16% to 92.5% of women did not provide a written and verbal informed consent before any procedure during labour and birth [22], while another study from Kenya documented that over 60% of women reported not consenting to newborn procedures [23]. In addition, few studies have reported challenges or barriers to the informed consent process in the maternal and newborn care area from health workers’ perspectives [24,25]. In Italy, according to the Physicians’ Deontological Code [26], consent request is a non-delegable physicians’ act. Consent may be given orally before the majority of procedures and written for selected major interventions (i.e., higher mortality risk interventions). However, in recent years, there has been an increasing number of medical malpractice lawsuits related to a lack of informed consent, with an increase in defensive practices (both from individuals and institutions), which has resulted in increased attention to the formal aspects of consent request, i.e., signing a form for informed consent [27,28,29,30]. Very few recent studies have explored practices related to consent request in Italy [15,16,27,28,29,30,31,32,33,34,35], with only a few focusing on childbirth practices [15,16,35]. 

This study aimed at exploring practices of consent request on nine key maternal and newborn clinical procedures during childbirth in a referral hospital in Northeast Italy, using both maternal and health workers’ perceptions.

## 2. Materials and Methods

### 2.1. Study Design

The study was designed as a cross-sectional observational study and is reported according to the STROBE (STrengthening the Reporting of OBservational studies in Epidemiology) [36] (Supplementary Table S1).

### 2.2. Study Setting and Population

This study was conducted between December 2016 and September 2018 in a large public referral university hospital in Northeast Italy [35].

All women giving birth during the study period and all clinical staff working in the maternal and neonatal wards directly with patients (i.e., midwives, nurses, obstetricians, neonatologists, undergraduate students and medical residents) were invited to participate. Exclusion criteria for women were: maternal death, perinatal death (including stillbirth), psychiatric or psychosocial problems with inability to fill in the questionnaire, age under 18 years old, language barriers (the questionnaire was available only in Italian) and refusal to participate. Exclusion criteria for health workers were: long absence from work (e.g., maternity leave or long-term sick leave) during the study period and refusal to participate. 

### 2.3. Data Collection and Variables

Data collection procedures have been reported elsewhere [35]. Briefly, data were collected using two self-administrated, anonymous and validated questionnaires in Italian [35] based on WHO standards [6]. The two questionnaires accounted, respectively, for 120 questions (women’s questionnaire) and 121 questions (health worker’s questionnaire). Detailed data collection procedures have been previously reported [35]. Data were entered into an electronic database following standard operating procedures; data quality was ensured by regular monitoring and interim analyses [35]. 

Out of the total questions in the two questionnaires, 27 indicators (Supplementary Table S2) pertaining to the fields “consent request”, “communication”, and “women autonomy” were selected for analysis in this study. In addition, eight variables on socio-demographic characteristics of both women and health workers and three variables for clinical characteristics of the women were used for the analysis.

### 2.4. Data Analysis

First, we performed a descriptive analysis looking at sample characteristics, frequency of consent requests, quality of information delivered by health workers prior to consent request, quality of the communication and women autonomy. All the above indicators were reported from women’s perspectives. Data were presented in tables and figures as absolute numbers and percentages. 

Additionally, the association between consent request on each one of the nine clinical procedures under study (Supplementary Table S2) and women socio-demographic characteristics (age, education, country of birth, nationality, occupational status) or clinical history (parity, emergency caesarean section [CS] and having a newborn admitted to neonatal intensive care unit [NICU]) was assessed using bivariate and multiple logistic regression models with consent request as the dependent variable.

Finally, organisational factors, availability of resources for consent request, and causes of ineffective communication were analysed from health workers’ perceptions and presented in tables and figures as absolute numbers and percentages.

All statistical tests were two-sided. A *p*-value less than 0.05 was considered statistically significant. Data were analysed using R version 3.6.1 (R Foundation for Statistical Computing, Vienna, Austria).

## 3. Results

### 3.1. Sample Characteristics

Overall, 1244 women and 105 health workers participated, with a questionnaire response rate of 52% for women and 80% for hospital staff (Supplementary Tables S3 and S4). There were no significant differences among the characteristics of responders and non-responders, except for a lower percentage of obstetricians among responders (*p* = 0.010) (Supplementary Table S5). All variables of interest had a very low missing rate (below 1.2%) (Supplementary Table S6). 

The sample characteristics and frequency of the nine key procedures under study are reported in Table 1. About half of the women had a bachelor’s or specialist degree (52.6%), were primiparous (52.9%), and were less than 35 years old (59.1%). About four out of five were employed (79.3%), while 15.5% were not born in Italy. The majority (88.6%) of health workers were female and over half (51.9%) had worked more than 10 years in maternal and newborn healthcare. Overall, most women (84.2%) were highly satisfied with the care received (score ≥ 7 out of 10). 

### 3.2. Frequency of Consent Requests (as Reported by Women)

**Figure 1:**
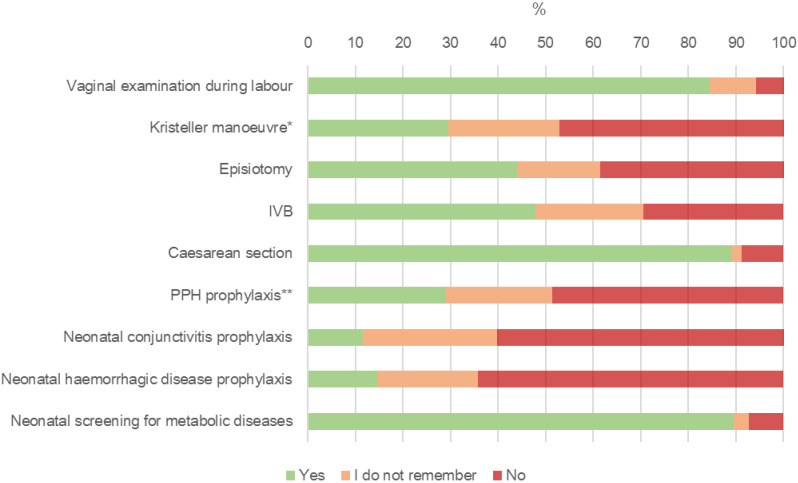
Consent request by type of clinical procedure according to women’s perception. Notes: All procedures were related to one or more WHO standards; * fundal pressure during second stage of labour as perceived by women; ** data generated only by women who had a vaginal birth or instrumental vaginal birth. Abbreviation: IVB = instrumental vaginal birth; PPH = maternal post-partum haemorrhage.

The women reported frequency of consent requests varied depending on the clinical procedure (Figure 1). In general, women consent was more frequently requested before maternal procedures than before neonatal procedures. For maternal procedures, the highest rate of consent request (89.1%) was reported for CS, with the lowest being for PPH prophylaxis (28.6%) after spontaneous birth or IVB. Women reported low rates of consent request (<15%) for two out of three neonatal procedures, with neonatal conjunctivitis prophylaxis (11.4%) presenting the lowest rate among all nine procedures evaluated. Additional details are provided in Supplementary Table S7.

### 3.3. Quality of Information Delivered by Health Workers Prior to Consent Requests

The quality of information delivered by health workers before requesting women’s consent was very heterogeneous across procedures (Table 2). Overall, while for CS 87.4% of women were informed both on risks and benefits and reasons for the procedures, this happened only in 18.6% of women who reported a Kristeller manoeuvre during the second stage of labour. Over 40% of women reported an incomplete information delivery before an episiotomy (42.7%) and before an IVB (41.2%).

### 3.4. Quality of the Communication and Women Autonomy

**Figure 2:**
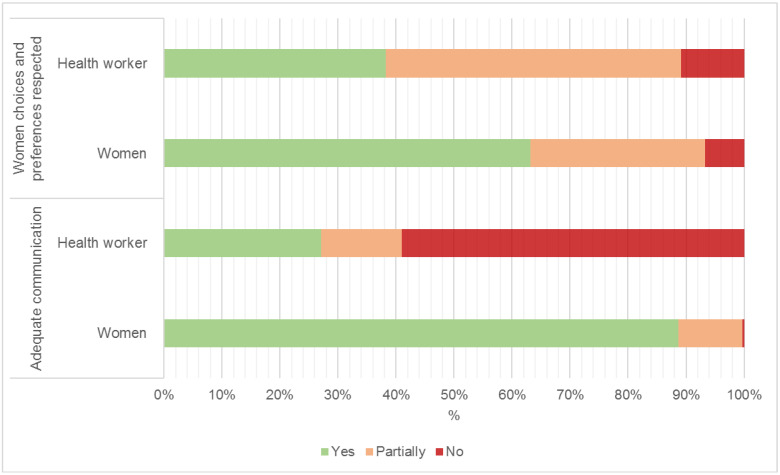
Communication and respect for women choices and preferences according to either women or health workers’ perceptions.

Almost three-quarters of women (74.8%) felt always/often adequately involved in the decision-making process and most of them (88.2%) reported adequate communication during care (Figure 2, Supplementary Table S8). In contrast, more than half (59.0%) of the health workers reported that communication with women and families was not adequate (Figure 2). A minority of health staff (10.5%) considered that women choices and preferences were not respected during care.

### 3.5. Women Characteristics Associated with Consent Request

Supplementary Tables S9 and S10 report the results of the univariate analyses, while Supplementary Table S11 presents the results of multiple logistic regression that explore associations between consent request as reported by women and socio-demographic characteristics and clinical history. Overall, only a few statistically significant associations were observed, with a variable pattern across different procedures. When considering maternal procedures, being ≥35 years old was negatively associated (OR 0.62, 95% IC 0.44–0.88, *p* = 0.007) with consent request for vaginal examinations during labour, while being employed and multiparous were positively associated with the consent request for the same procedure (OR 2.19, 95% IC 1.46–3.25, *p* < 0.001 and OR 1.75, 95% IC 1.23–2.5, *p* = 0.002, respectively). Receiving an emergency CS was negatively associated with consent request (OR 0.09, 95% IC 0.01–0.33, *p* = 0.002) for that procedure.

Regarding neonatal procedures, women high education (i.e., bachelor’s degree/specialist degree) was negatively associated with consent request (OR 0.6, 95% IC 0.42–0.91, *p* = 0.015) for neonatal conjunctivitis prophylaxis and women foreign nationality was positively associated with the consent request for the same procedure (OR 2.64, 95% IC 1.14–6.7, *p* = 0.030). The consent request for the neonatal screening for metabolic diseases was negatively associated with women place of birth (OR 0.4, 95% IC 0.21–0.78, *p* = 0.005) and NICU admission (OR 0.28, 95% IC 0.18–0.45, *p* < 0.001). No other significant associations were found.

### 3.6. Health Workers’ Perspectives on Organisational Factors and Availability of Resources for Consent Request

Among the 105 health workers participating in the survey, about two-thirds reported the availability of adequate protocols (67.6%) and standard forms (78.1%) for consent request. However, less than one-third (31.4%) referred to the presence of adequate in-service hospital training and supportive supervision in this field. One out of five (20.0%) reported the existence of mechanisms to identify an event where women could not express an informed choice, while about half (50.5%) were unsure about their existence (Table 3). 

### 3.7. Health Workers’ Perspectives on Causes of Ineffective Communication

**Figure 3:**
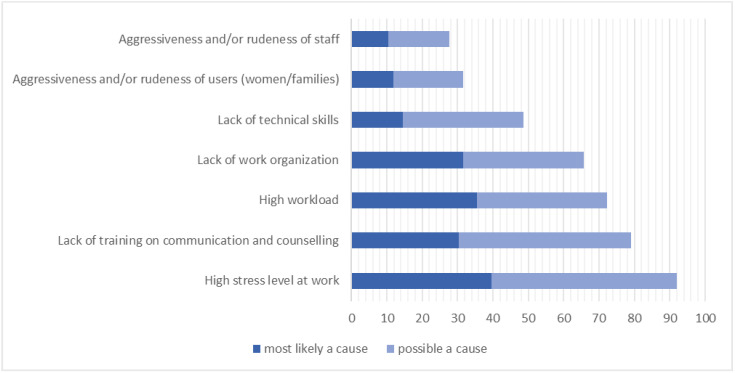
Health workers’ perspectives on causes of ineffective communication with women and families during childbirth (N = 76 *). Notes: * only health staff who judged communication with women/families partially adequate or not adequate answered the following question: “how much do you think that the following factors, may be the causes of ineffective communication with women and their families in your facility?” which had pre-determined answer categories.

The main causes of ineffective communication with women and families according to health workers’ perspectives were: high stress levels at work (39.5%), high work load (35.5%) and lack of work organisation (31.6%) (Figure 3 and Supplementary Table S12).

## 4. Discussion

This is the first study exploring consent request practices around the time of childbirth in Italy according to both women and health workers’ perspectives for nine key clinical procedures. Overall, the study revealed that, according to women as key receivers, consent request frequency varied for most maternal procedures under study (range 28.6–89.1%), except for vaginal examination during labour and CS, and information provided by health workers before requests was most often (range 18.6–87.4%) incomplete. Early neonatal care procedures had even lower rates of consent request (lower than 15%, except for neonatal screening for metabolic diseases). Results of the multivariate analyses suggested that the lack of informed consent is not specifically linked to any pattern of specific women characteristics and all women are potentially exposed. Health workers reported the availability of protocols and standard forms for consent request, but a lack of training and supportive supervision in the field. Interestingly, they rated quality in communication and women autonomy lower than women. Despite being based on people reports and not direct observations of care, these findings highlight the urgent need for quality improvement on consent request practices during childbirth and more research on the field in Italy.

Our findings confirm and expand previous evidence [14,15,16,17,18,37,38,39,40,41,42,43,44,45,46,47,48]. The lack of informed consent is one of the typologies of mistreatment during childbirth [49,50]. Other authors have recently reported a frequent women’s perception of a lack of consensus request for maternal procedures during childbirth in high-income countries of the WHO European region [15,16,17,47,48]. In addition, there is limited information from both low- and high-income countries about respectful care measures and consent request practices for newborn procedures [51].

Overall existing literature suggests that the childbirth setting may add specific challenges to consent requests. The time around childbirth can be challenging for an appropriate consent request, both from women and health professionals’ perspectives. Many mothers experience birth only once in life, thus previous experience is limited. Effective communication can be challenged by many factors, including: the limited time available for discussion in emergency situations (e.g., emergency caesarean section, foetal and/or maternal complications), the possible influence of women’s pain, fatigue, and emotions and by the availability of standardised documents clarifying risks and benefits of procedures [18,37,38,39]. When interpreting our results, therefore, we believe that it is important to avoid blaming health professionals for “low rates” of consent requests. As highlighted by a recent metanalysis [52], the factors underlying the attitudes and behaviours of health professionals can have causes at different levels, such as societal level (cultural beliefs and social context), organisational level (workload, public or private sector, presence of supportive supervision) and individual level (training, personal motivation, stress and expectations). Indeed, this study suggested that the existence of protocols is not enough, and a lack of training and supervision, high stress and high workload might act as barriers to effective consent request practices. Other studies identified additional barriers to effective and efficient consent request in Italy, such as consent forms written in technical jargon style and too complex for the average patient [31], the “inability” of patients to understand the information given [34], and information not even read by caregivers [32]. More research is needed to further document the underlying causes of ineffective consent request.

The request for consent is just one of the steps in a more complex path of the decision-making process. It should be accompanied by a trustful relationship between professionals and patients, transparent communication of the best available evidence, discussion of the individual’s preferences, and shared decision making [53,54]. Italian law currently recognises the patient’s right to have adequate time to discuss preferences with physicians and requires that facilities develop an organisational structure to ensure adequate information about patients and adequate competencies among staff [28,29,44,55]. However, this may be difficult to achieve in practice, as suggested by this study. In routine practice, there is a lack of consensus—and perhaps, experience—on how better to accomplish this goal, especially in settings characterised by high patient workload or in emergency situations, such as emergency CS or when patients refuse the care proposed [54,56], as frequently observed in recent times, during the COVID-19 pandemic, in respect to new vaccines and novel prevention and treatments protocols [57].

Effective solutions for improving consensus requests and increasing patient understanding and recall of information given involve multiple aspects of organisation of care. A review published in the New England Journal of Medicine [1] proposed a set of strategies linked to health system organisation, e.g., the use of technology or creative solutions to present information, involving families/trusted friends in discussion, and alternative assessments of capacities and training of health professionals. In addition, a recent systematic review of 73 studies found that multimedia tools (interactive and non-interactive) seem desirable for improving consent request practices since they might have a higher impact than information videos only [58]. A community-based study showed that group counselling may be another valid option, in terms of logistics and easy delivery, in low-income community settings when compared to individual counselling [59]. 

In particular, during obstetric emergencies (i.e., IVB, CS and even more emergency CS), it may be impossible to disclose all potential risks and complications of those procedures without bias (“framing effect” when clinicians intentionally may frame information provided toward the outcome preferred by them) that might hinder women’s autonomy [24,37,41,46,60]. Therefore, future research should explore the effectiveness of alternative ways to provide informed consent for emergency procedures that may include timely consultation and better patient information during antenatal care courses with the support of videos, simulations and multimedia tools. 

Limitations of this study include: informed consent practices were not assessed by direct observation or triangulation of data with hospital records, being potentially exposed to recall bias or even to the “halo effect” (unconscious judgments of the target dimension influenced by likeable personality, or some specific desirable experience lived by subject’s) [61]. Moreover, no specific questions were asked about the timing of requests. Most mothers were highly satisfied with the overall care received, and therefore consent request frequency may be overestimated by them. Since our questionnaire was only in Italian, we were not able to study the experience of non-Italian speaking women who may have different experiences in the decision-making process participation due to linguistic barriers or other factors. Results from this study cannot be generalised to settings different from referral hospitals in high-income countries. 

Further research should investigate practices on informed consent in other settings, ideally including triangulation of data from multiple sources (if possible, direct observation). This study was further scaled up in eight additional facilities in 2019 [15,16,62,63], and the results will be reported in future publications. Most importantly, more studies are urgently needed to identify which interventions or their combination are effective to improve consent request practices during childbirth.

## 5. Conclusions

The study findings confirm and expand previous evidence showing that gaps in consent request are frequent. However, while there is a lack of consensus on how to better improve this practice in routine women-centred care, it is important to recognise the possible role of systemic factors (e.g., low quality of standard forms, timing of requests). More research and action are needed to investigate practices and to explore the impact of different strategies of improvement. 

## Figures and Tables

**Table 1:** Characteristics of the sample, clinical procedures and overall women’s satisfaction with care received.

**Women**	***n*** **(N = 1244)**	**%**
Age < 35 years old	735	59.1
Age ≥ 35 years old	509	40.9
Bachelor’s degree/specialist degree	655	52.6
Italian nationality	1124	90.3
Born in Italy	1051	84.5
Employed women	987	79.3
Primiparous	658	52.9
Single pregnancy	1223	98.3
Newborn NICU admission	145	11.7
Women highly satisfied with care received (score ≥ 7)	1047	84.2
**Frequency of nine key procedures under study ^1^**	**N**	**%**
Maternal care		
Vaginal examination during labour	1094	87.9
Kristeller manoeuvre ^2^	130	10.5
Episiotomy	166	13.3
Instrumental vaginal birth	120	9.6
Caesarean section	285	22.9
PPH prophylaxis ^3^	959	77.1
Neonatal care		
Neonatal conjunctivitis prophylaxis	1244	100
Neonatal haemorrhagic disease prophylaxis (Vitamin K)	1244	100
Neonatal screening for metabolic diseases	1244	100
**Health workers**	***n*** **(N = 105)**	**%**
Gender: female	93	88.6
Profession		
Midwife	36	34.3
Nurse	27	25.7
Obstetrician	15	14.3
Neonatologist	9	8.6
Undergraduate students/medical residents	18	17.1
More than 10 years of work in maternal and newborn care	54	51.9

Notes: ^1^ All procedures were reported according to mothers’ perception; ^2^ fundal pressure perceived by women during second stage of labour; ^3^ data generated only by women that had a vaginal or instrumental vaginal birth. Abbreviation: NICU = neonatal intensive care unit; PPH = maternal post-partum haemorrhage.

**Table 2:** Quality of information delivered by health workers * prior to consent requests.

	Women Informed Both on Risks and Benefits and Reasons for the Procedures *n* (%)	Incomplete Information ^2^ *n* (%)	No Information for Both Practices *n* (%)	Not Remembered by Women for Both Practices *n* (%)
Kristeller manoeuvre ^1^ (N = 129)	24 (18.6)	50 (38.8)	39 (30.2)	16 (12.4)
Episiotomy (N = 166)	65 (39.2)	71 (42.7)	22 (13.3)	8 (4.8)
Instrumental vaginal birth (N = 119)	45 (37.8)	49 (41.2)	13 (10.9)	12 (10.1)
Caesarean sections (N = 285)	249 (87.4)	34 (11.8)	1 (0.4)	1 (0.4)

Notes: * Hospital staff expected practices (“provide information on risks and benefits” and “explain reasons” before each procedure) were assessed in two different questions for each procedure of interest; ^1^ fundal pressure perceived by women during second stage of labour; ^2^ women who reported that information on either risks and benefits or reasons for the procedures were (i) not delivered by health workers or (ii) not remembered by women.

**Table 3:** Health workers’ perspectives on organizational factors and availability of resources for consent requests (N = 105).

	Yes *n* (%)	Partially Yes *n* (%)	I Do Not Remember/I Am Not Sure *n* (%)	No *n* (%)
Availability of adequate protocols for consent	71 (67.6)	16 (15.2)	8 (7.6)	7 (6.7)
Availability of adequate standard consent forms	82 (78.1)	19 (18.1)	2 (1.9)	0
Presence of adequate in-service hospital training and supportive supervision for consent request	33 (31.4)	35 (33.3)	7 (6.7)	27 (25.7)
Existence of mechanisms to identify an event where women could not express an informed choice	21 (20.0)	8 (7.6)	53 (50.5)	15 (14.3)

## Data Availability

All relevant data are provided in the paper. Additional details can be provided by contacting corresponding author on a reasonable request.

## Supplementary Materials

The following supporting information can be downloaded at: https://www.mdpi.com/article/10.3390/ijerph19127166/s1, Table S1: STROBE Checklist for reports of cross-sectional studies; Table S2. Variables of interest; Table S3. Flow diagram of women; Table S4. Flow diagram of health workers; Table S5. Characteristics of missing cases; Table S6. Missing variable; Table S7. Consent request by type of clinical procedure according to women’s perception; Table S8. Women’s perception of other aspects of quality of care related to consent request (N = 1244); Table S9. Association between consent request as reported by women and socio-demographic characteristics; Table S10. Association between consent request as reported by women and clinical history; Table S11. Association between consent request as reported by women and socio-demographic characteristics/clinical history: results of multiple logistic regression; Table S12. Health workers’ perspectives on possible causes of ineffective communication with women/families during childbirth (N = 76 *).
