# Supplementary material for: Quality of Informed Consent Practices around the Time of Childbirth: A Cross-Sectional Study in Italy
Source: Int J Environ Res Public Health. 2022 Jun 10;19(12):7166. doi: 10.3390/ijerph19127166 (PMC9222941; doi:10.3390/ijerph19127166)
Supplement: Supplementary file 1 [file ijerph-19-07166-s001.zip › ijerph-1727348-supplementary.pdf]

Quality of informed consent practices around the time of childbirth: a cross-sectional study in Italy

Supplementary file

Table of Contents

Supplementary Table S1. STROBE Checklist for reports of cross-sectional studies.........2

Supplementary Table S2. Variables of interest.........4

Supplementary Table S3. Flow diagram of women.........5

Supplementary Table S4. Flow diagram of health workers.........6

Supplementary Table S5. Characteristics of missing cases.........7

Supplementary Table S6. Missing variable.........8

Supplementary Table S7. Consent request by type of clinical procedure according to women perception.........9

Supplementary Table S8. Women's perception of other aspects of quality of care related to consent request (N=1244).........10

Supplementary Table S9. Association between consent request as reported by women and socio-demographic characteristics.........11

Supplementary Table S10. Association between consent request as reported by women and clinical history.........12

Supplementary Table S11. Association between consent request as reported by women and socio-demographic characteristics / clinical history: results of multiple logistic regression.........13

Supplementary Table S12. Health worker's perspectives on possible causes of ineffective communication with women/families during childbirth (N=76*).........15

Supplementary Table S1. STROBE Checklist for reports of cross-sectional studies

	Item No.	Recommendation	Page No.
**Title and abstract**	1	(*a*) Indicate the study's design with a commonly used term in the title or the abstract	1-2
		(*b*) Provide in the abstract an informative and balanced summary of what was done and what was found	2
**Introduction**			
Background/rationale	2	Explain the scientific background and rationale for the investigation being reported	3
Objectives	3	State specific objectives, including any prespecified hypotheses	4
**Methods**			
Study design	4	Present key elements of study design early in the paper	4
Setting	5	Describe the setting, locations, and relevant dates, including periods of recruitment, exposure, follow-up, and data collection	4
Participants	6	(*a*) *Cross-sectional study*—Give the eligibility criteria, and the sources and methods of selection of participants	4
Variables	7	Clearly define all outcomes, exposures, predictors, potential confounders, and effect modifiers. Give diagnostic criteria, if applicable	4-5
Data sources/ measurement	8*	For each variable of interest, give sources of data and details of methods of assessment (measurement). Describe comparability of assessment methods if there is more than one group	4-5
Bias	9	Describe any efforts to address potential sources of bias	5
Study size	10	Explain how the study size was arrived at	5
Quantitative variables	11	Explain how quantitative variables were handled in the analyses. If applicable, describe which groupings were chosen and why	5
Statistical methods	12	(*a*) Describe all statistical methods, including those used to control for confounding	5
		(*b*) Describe any methods used to examine subgroups and interactions	5
		(*c*) Explain how missing data were addressed	5
		(*d*) *Cross-sectional study*—If applicable, describe analytical methods taking account of sampling strategy	5
		(*e*) Describe any sensitivity analyses	5
**Results**			

Participants	13*	(a) Report numbers of individuals at each stage of study—eg numbers potentially eligible, examined for eligibility, confirmed eligible, included in the study, completing follow-up, and analysed	5-7
		(b) Give reasons for non-participation at each stage	5-7
		(c) Consider use of a flow diagram	Page 5 and Supplementary Table 3 and 4
Descriptive data	14*	(a) Give characteristics of study participants (eg demographic, clinical, social) and information on exposures and potential confounders	5-6
		(b) Indicate number of participants with missing data for each variable of interest	5-6 and Supplementary Table 6
Outcome data	15*	*Cross-sectional study*—Report numbers of outcome events or summary measures	7
Main results	16	(*a*) Give unadjusted estimates and, if applicable, confounder-adjusted estimates and their precision (eg, 95% confidence interval). Make clear which confounders were adjusted for and why they were included	7-11
		(*b*) Report category boundaries when continuous variables were categorized	7-11
		(*c*) If relevant, consider translating estimates of relative risk into absolute risk for a meaningful time period	7-11
Other analyses	17	Report other analyses done—eg analyses of subgroups and interactions, and sensitivity analyses	7-11
**Discussion**			
Key results	18	Summarise key results with reference to study objectives	12
Limitations	19	Discuss limitations of the study, taking into account sources of potential bias or imprecision. Discuss both direction and magnitude of any potential bias	14
Interpretation	20	Give a cautious overall interpretation of results considering objectives, limitations, multiplicity of analyses, results from similar studies, and other relevant evidence	13-14
Generalisability	21	Discuss the generalisability (external validity) of the study results	13-14
**Other information**			
Funding	22	Give the source of funding and the role of the funders for the present study and, if applicable, for the original study on which the present article is based	15

*Give information separately for cases and controls in case-control studies and, if applicable, for exposed and unexposed groups in cohort and cross-sectional studies

Supplementary Table S2. Variables of interest

**Women's questionnaire**	**16 indicators**<br>- Frequency of consent request for nine key clinical procedures<br>*Maternal care* *****<br>Vaginal examination during labour<br>Kristeller manoeuvre<br>Episiotomy<br>Instrumental vaginal birth<br>Caesarean section<br>Post-partum haemorrhage prophylaxis<br>*Neonatal care*<br>Neonatal conjunctivitis prophylaxis<br>Neonatal haemorrhagic disease prophylaxis [Vitamin K]<br>Neonatal screening for metabolic diseases);<br>- Risks, benefits and reasons (information delivery by health worker's before requesting women's consent) for: Kristeller manoeuvre, Episiotomy, Instrumental vaginal birth, and Caesarean section<br>- Adequate communication<br>- Respect to requests and needs<br>- Women overall satisfaction with care received (measured on a Likert scale from 1 minimum to 10 maximum satisfaction)
**Health worker's questionnaire**	**11 indicators**<br>- Presence of resources (e.g protocols, forms for consent request)<br>- Organisational factors related with consent request (e.g. availability of training)<br>- Respect for women choices<br>- Adequate communication with women and families<br>- Reasons for inadequate communication**

Notes: * according to women mode of birth; ** all indicators were related with one or more WHO Standards [6] except for this one.

Supplementary Table S3. Flow diagram of women

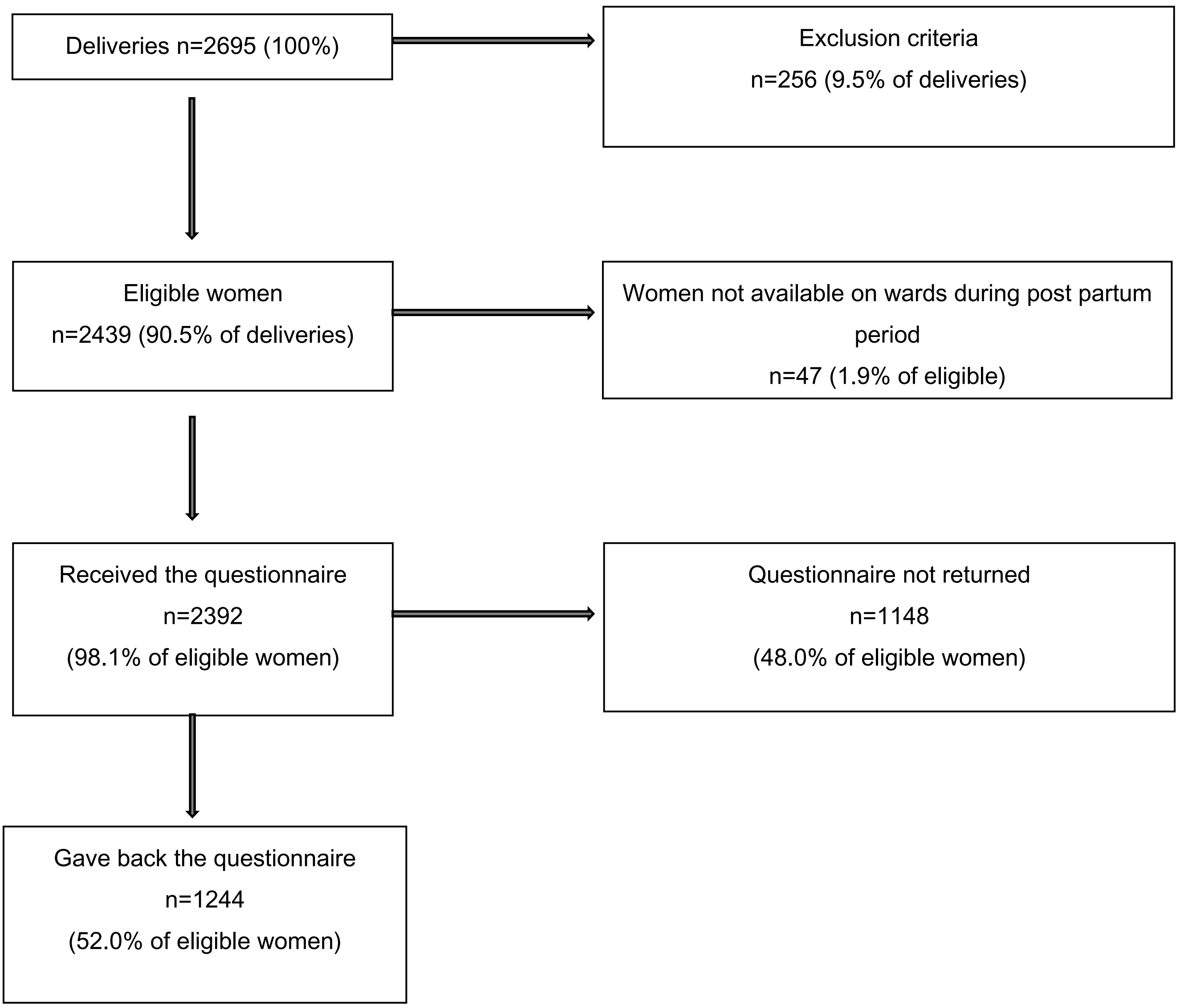

Supplementary Table S4. Flow diagram of health workers

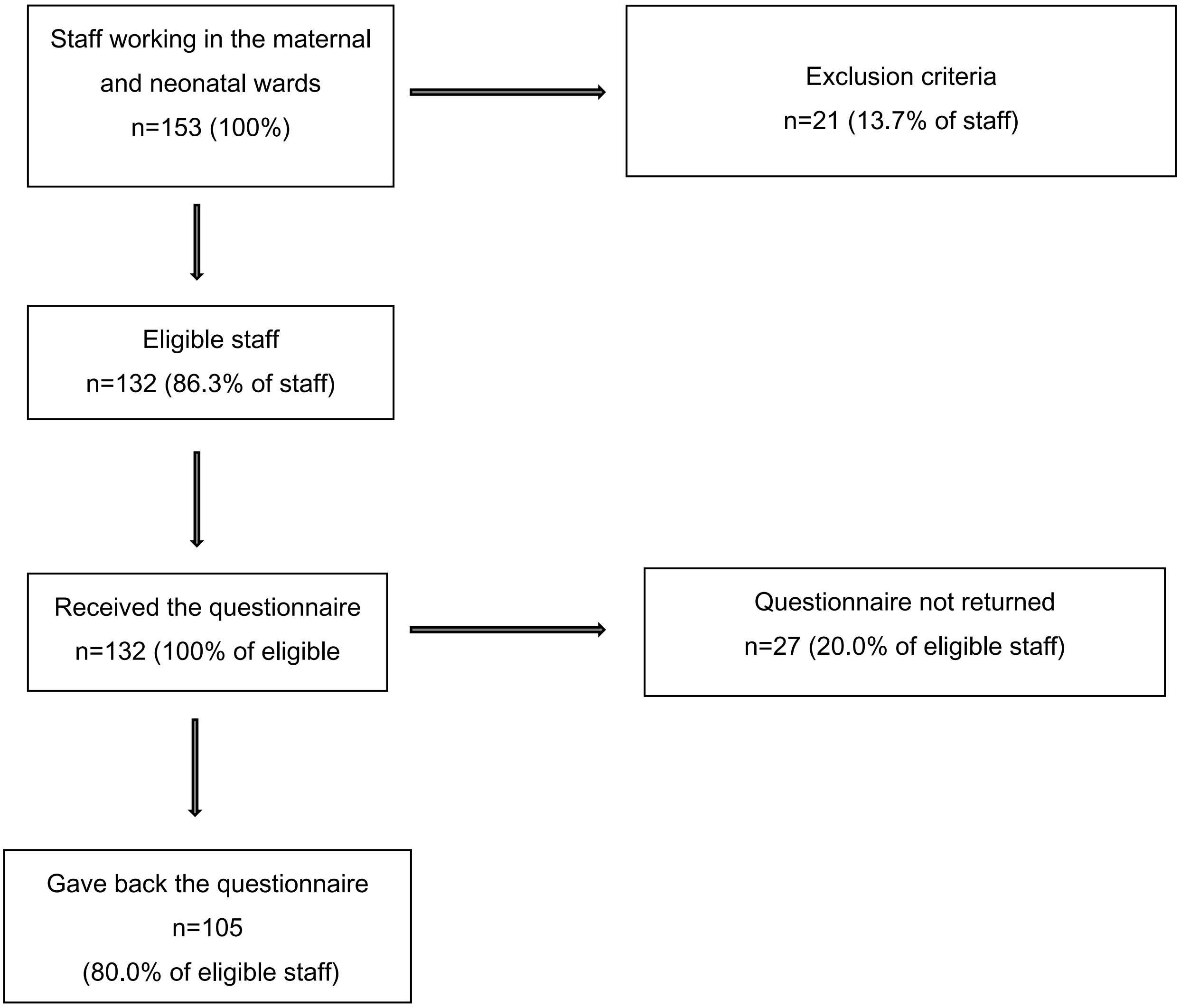

Supplementary Table S5. Characteristics of missing cases

	Enrolled<br>n (%)	Missing cases<br>n (%)	p value
**Women survey**	**N=1244**	**N=1148**	
Primiparous	658 (52.9)	642 (55.9)	0.12
Multiparous	586 (47.1)	506 (44.1)	0.12
Normal vaginal birth	839 (67.4)	790 (68.8)	0.47
Instrumental vaginal birth	120 (9.6)	111 (90.3)	0.93
Cesarean section	285 (22.9)	247 (21.5)	0.39
Neonatal intensive care unit	145 (11.7)	134 (11.7)	0.16
Multiple pregnancy	21 (1.7)	26 (2.3)	0.31
**Health workers survey**	**N=105**	**N=27**	
Female professionals	93 (88.6)	21 (77.8)	0.25
Midwives	36 (34.3)	5 (18.5)	0.17
Nurses	27 (25.7)	6 (22.2)	0.90
Obstetricians	15 (14.3)	10 (37.0)	**0.01**
Neonatologists	9 (8.6)	1 (3.7)	0.65
Obstetrician residents	6 (5.7)	5 (18.5)	0.07

Supplementary Table S6. Missing variable

	Total	Missing data	% Missing
**Women**			
Age	1244	0	0
Education	1244	7	0.6
Nationality	1244	9	0.7
Born in Italy	1244	6	0.5
Employed women	1244	6	0.5
Primiparous	1244	0	0
Single pregnancy	1244	0	0
Newborn NICU admission	1244	2	0.2
**Procedures reported by women**			
Vaginal examination during labour	1098	4	0.4
Kristeller manoeuvre [1]	130	1	0.8
Episiotomy	166	0	-
Instrumental vaginal birth	120	1	0.8
Caesarean section	285	0	-
PPH prophylaxis [2]	959	11	1.1
Neonatal conjunctivitis prophylaxis	1244	5	0.4
Neonatal haemorrhagic disease prophylaxis (Vitamin K)	1244	6	0.5
Newborn screening for metabolic diseases	1244	2	0.2
**Women's overall satisfaction with care received**	1244	14	1.1
**Health workers**			
Gender	105	0	0
Profession	105	0	0
Years of work in maternal and newborn care	105	1	1.0

Notes: All procedures were related with one or more WHO Standards; [1] fundal pressure performed during second stage of labour as perceived by women; [2] data generated only by women that had a vaginal birth or instrumental vaginal birth. Abbreviation: NICU = neonatal intensive care unit; PPH = maternal post-partum haemorrhage.

Supplementary Table S7. Consent request by type of clinical procedure according to women perception

Procedures	Yes n (%)	I do not remember n (%)	No n (%)	Missing n (%)
Vaginal examination during labour (N=1098)	924 (84.2)	106 (9.7)	64 (5.8)	3 (0.03)
Kristeller manoeuvre [1] (N=130)	38 (29.2)	30 (23.1)	61 (46.9)	1 (0.8)
Episiotomy (N=166)	73 (44.0)	29 (17.5)	64 (38.6)	0
Instrumental vaginal birth (N=120)	57 (47.5)	27 (22.5)	35 (29.2)	1 (0.8)
Caesarean section (N=285)	245 (89.1)	6 (2.1)	25 (8.8)	0
PPH prophylaxis [2] (N=959)	274 (28.6)	213 (22.2)	461 (48.1)	11 (1.1)
Neonatal conjunctivitis prophylaxis (N=1244)	142 (11.4)	349 (28.1)	748 (60.1)	5 (0.4)
Neonatal haemorrhagic disease prophylaxis (N=1244)	180 (14.5)	262 (21.1)	796 (64.0)	6 (0.4)
Neonatal screening for metabolic diseases (N=1244)	1112 (89.4)	40 (3.2)	90 (7.2)	2 (0.2)

Notes: All procedures were related with one or more WHO Standards; [1] fundal pressure during second stage of labour as perceived by women; [2] data generated only by women who had a vaginal birth or instrumental vaginal birth.
Abbreviation: PPH = maternal post-partum haemorrhage.

Supplementary Table S8. Women's perception of other aspects of quality of care related to consent request (N=1244)

	Always<br>n (%)	Often<br>n (%)	Sometimes<br>n (%)	Rarely<br>n (%)	Never<br>n (%)
Experienced adequate communication	693 (55.7)	404 (32.5)	119 (9.6)	18 (1.4)	4 (0.3)
Felt adequately involved in the decision-making process	474 (38.1)	457 (36.7)	215 (17.3)	64 (5.1)	24 (1.9)
Felt coerced into accepting care suggested	36 (2.9)	47 (3.8)	161 (12.9)	212 (17.0)	782 (62.9)

Supplementary Table S9. Association between consent request as reported by women and socio-demographic characteristics

Clinical procedure for which women received a consent request*	Women characteristics									
	Age ≥35		High education		Foreign nationality		Born abroad		Employed women	
	OR (95% CI)	p-value	OR (95% CI)	p-value	OR (95% CI)	p-value	OR (95% CI)	p-value	OR (95% CI)	p-value
Vaginal examination during labour	0.74 (0.53-1.03)	0.077	0.78 (0.52-1.16)	0.2440	1.49 (0.81-3.01)	0.185	1.40 (0.87-2.37)	0.185	1.69 (1.15-2.43)	**0.006**
Kristeller manoeuvre	0.93 (0.42-2.03)	0.862	1.21 (0.50-3.18)	0.6792	0.89 (0.19-3.28)	0.868	0.77 (0.23-2.17)	0.635	2.88 (0.90-12.86)	0.108
Episiotomy	0.79 (0.41-1.48)	0.464	0.69 (0.31-1.56)	0.373	0.55 (0.17-1.59)	0.286	0.48 (0.19-1.13)	0.106	1.64 (0.75-3.79)	0.226
Instrumental vaginal birth	1.01 (0.49-2.09)	0.973	0.62 (0.26-1.44)	0.265	0.81 (0.15-3.81)	0.783	0.45 (0.13-1.32)	0.159	0.64 (0.24-1.64)	0.352
Caesarean section	1.05 (0.50-2.26)	0.891	0.54 (0.15-1.45)	0.267	0.84 (0.30-3.01)	0.766	1.13 (0.44-3.48)	0.812	1.99 (0.82-4.50)	0.110
PPH prophylaxis	0.93 (0.69-1.24)	0.606	0.99 (0.71-1.37)	0.932	0.60 (0.33-1.01)	0.067	0.83 (0.55-1.25)	0.389	0.94 (0.67-1.33)	0.732
Neonatal conjunctivitis prophylaxis	0.82 (0.57-1.18)	0.287	0.64 (0.44-0.95)	**0.022**	2.55 (1.56-4.06)	**<0.001**	1.86 (1.20-2.81)	**0.004**	0.77 (0.51-1.17)	0.207
Neonatal haemorrhagic disease prophylaxis (Vit K)	0.88 (0.64-1.22)	0.454	0.96 (0.67-1.40)	0.824	1.35 (0.80-2.19)	0.243	1.13 (0.73-1.72)	0.563	1.06 (0.72-1.60)	0.769
Neonatal screening for metabolic diseases	1.08 (0.74-1.57)	0.697	0.83 (0.52-1.28)	0.412	0.55 (0.33-0.97)	**0.029**	0.45 (0.30-0.70)	**<0.001**	1.61 (1.05-2.41)	**0.024**

Notes: * All procedures were related with one or more WHO Standards

Abbreviation: PPH = maternal post-partum haemorrhage.

Supplementary Table S10. Association between consent request as reported by women and clinical history

**Clinical procedure for which women received a consent request ***	**Women clinical history**					
	**Multiparous women**		**Emergency caesarean section**		**NICU admission**	
	OR (95% CI)	p-value	OR (95% CI)	p-value	OR (95% CI)	p-value
Vaginal examination during labour	1.58 (1.13-2.22)	**0.008**	0.86 (0.54-1.40)	0.521	1.39 (0.77-2.73)	0.306
Kristeller manoeuvre	0.87 (0.34-2.04)	0.750	0.00 (NA-NA)	0.992	0.78 (0.17-2.80)	0.723
Episiotomy	0.74 (0.36-1.49)	0.406	0.00 (NA-NA)	0.987	0.72 (0.25-1.88)	0.507
Instrumental vaginal birth	1.03 (0.45-2.34)	0.950	- **	-	0.65 (0.19-2.07)	0.473
Caesarean section	3.34 (1.41-9.24)	**0.011**	0.09 (0.01-0.30)	**0.001**	0.77 (0.35-1.85)	0.541
PPH prophylaxis	1.11 (0.83-1.46)	0.483	- **	-	1.43 (0.86-2.32)	0.159
Neonatal conjunctivitis prophylaxis	0.77 (0.54-1.10)	0.157	1.32 (0.81-2.09)	0.243	1.29 (0.75-2.10)	0.332
Neonatal haemorrhagic disease prophylaxis (Vitamin K)	0.95 (0.69-1.31)	0.775	0.86 (0.52-1.36)	0.525	0.99 (0.59-1.60)	0.984
Neonatal screening for metabolic diseases	1.43 (0.99-2.08)	0.058	0.48 (0.31-0.77)	**0.001**	0.25 (0.16-0.38)	**<0.001**

Notes: * All procedures were related with one or more WHO Standards; ** women receiving an emergency caesarean section do not answer questions regarding instrumental vaginal births nor PPH prophylaxis.

Abbreviation: NICU = neonatal intensive care unit; PPH = maternal post-partum haemorrhage.

Supplementary Table S11. Association between consent request as reported by women and socio-demographic characteristics / clinical history: results of multiple logistic regression

Maternal procedures*												
	Vaginal examination during labour N=1090		Kristeller manoeuvre N=128		Episiotomy N=165		Instrumental vaginal birth N=119		Caesarean section N=281		PPH prophylaxis N=946	
	OR	p value	OR	p value	OR	p value	OR	p value	OR	p value	OR	p value
Age ≥35	0.62 (0.44-0.88)	**0.007**	0.91 (0.4-2.01)	0.815	0.73 (0.38-1.41)	0.353	1 (0.46-2.17)	0.991	0.71 (0.3-1.66)	0.430	0.88 (0.65-1.19)	0.414
High education**	0.76 (0.5-1.13)	0.192	1.4 (0.55-3.82)	0.495	0.66 (0.28-1.52)	0.326	0.52 (0.2-1.32)	0.177	0.49 (0.13-1.39)	0.216	0.98 (0.71-1.38)	0.926
Foreign nationality	1.31 (0.5-3.41)	0.572	1.58 (0.19-15.65)	0.672	1.53 (0.27-9.66)	0.633	3.33 (0.35-38.22)	0.299	0.3 (0.01-2.86)	0.335	0.47 (0.22-1.01)	0.055
Born abroad	1.37 (0.69-3.03)	0.405	0.8 (0.11-3.77)	0.792	0.43 (0.09-1.59)	0.231	0.2 (0.03-0.94)	0.063	5.13 (0.84-102.03)	0.145	1.21 (0.67-2.12)	0.522
Employed women	2.19 (1.46-3.25)	**<0.001**	2.94 (0.86-13.78)	0.117	1.28 (0.53-3.18)	0.587	0.47 (0.16-1.31)	0.157	3.4 (1.12-10.16)	**0.028**	0.91 (0.64-1.31)	0.619

Multiparous women	1.75 (1.23-2.5)	**0.002**	0.91 (0.35-2.27)	0.845	0.75 (0.36-1.55)	0.439	0.95 (0.39-2.29)	0.901	2.31 (0.9-6.82)	0.101	1.13 (0.84-1.51)	0.416
Emergency CS ***	0.87 (0.54-1.46)	0.592	-	-	-	-	-	-	0.09 (0.01-0.33)	**0.002**	-	-
NICU admission	1.58 (0.86-3.14)	0.165	1.02 (0.2-4.13)	0.977	0.74 (0.25-2.05)	0.570	0.64 (0.17-2.22)	0.490	1.22 (0.5-3.22)	0.677	1.43 (0.86-2.33)	0.161

Neonatal procedures*

	Neonatal conjunctivitis prophylaxis N=1233		Neonatal hemorrhagic disease prophylaxis (Vitamin K) N=1233		Neonatal screening for metabolic diseases N=1236	
	OR	p value	OR	p value	OR	p value
Age ≥35	0.90 (0.61-1.32)	0.602	0.89 (0.63-1.25)	0.502	1.00 (0.67-1.50)	0.987
High education**	0.61 (0.42-0.91)	**0.015**	0.94 (0.65-1.38)	0.757	0.84 (0.52-1.32)	0.463
Foreign nationality	2.64 (1.14-6.7)	**0.030**	1.52 (0.67-3.63)	0.328	1.38 (0.62-3.02)	0.422
Born abroad	0.94 (0.4-1.96)	0.885	0.89 (0.41-1.74)	0.751	0.4 (0.21-0.78)	**0.005**
Employed women	0.97 (0.62-1.55)	0.892	1.17 (0.77-1.82)	0.462	1.37 (0.86-2.15)	0.181
Multiparous women	0.74 (0.51-1.07)	0.112	0.93 (0.67-1.3)	0.686	1.45 (0.98-2.17)	0.066
Emergency CS	1.21 (0.72-1.98)	0.456	0.86 (0.51-1.38)	0.539	0.77 (0.47-1.27)	0.289
NICU admission	1.16 (0.65-1.95)	0.601	1.06 (0.62-1.73)	0.823	0.28 (0.18-0.45)	**<0.001**

Note: *All procedures were related with one or more WHO Standards; ** Bachelor's degree / Specialist degree; *** Emergency CS was used as independent variable for i) Kristeller manoeuvre and episiotomy procedures as only one woman received both of those procedures before the emergency CS; ii) instrumental vaginal birth and PPH prophylaxis procedures because women receiving an emergency caesarean section do not answer questions regarding them.

Abbreviation: CS = caesarean section; NICU = neonatal intensive care unit; PPH = maternal post-partum haemorrhage

Supplementary Table S12. Health worker's perspectives on possible causes of ineffective communication with women/families during childbirth (N=76*)

Causes	Not a cause n (%)	Less likely a cause n (%)	Possibly a cause n (%)	Most likely a cause n (%)	Missing n (%)
High stress level at work	2 (2.6)	4 (5.3)	40 (52.6)	30 (39.5)	0 (0.0)
Lack of training on communication and counselling	1 (1.3)	15 (19.7)	37 (48.7)	23 (30.3)	0 (0.0)
High workload	0 (0.0)	21 (27.6)	28 (36.8)	27 (35.5)	0 (0.0)
Lack of work organization	3 (3.9)	22 (28.9)	26 (34.2)	24 (31.6)	1 (1.3)
Lack of technical skills	13 (17.1)	26 (34.2)	26 (34.2)	11 (14.5)	0 (0.0)
Aggressiveness and/or rudeness of users (women/families)	5 (6.9)	45 (59.2)	15 (19.7)	9 (11.8)	2 (2.6)
Aggressiveness and/or rudeness of staff	11 (14.5)	43 (56.6)	13 (17.1)	8 (10.5)	1 (1.3)

Notes: * only health staff who judged communication with women/families partially adequate or not adequate answered the following question: "how much do you think that the following factors, may be the causes of ineffective communication with women and their families in your facility?" which had pre-determined answer categories.
